# The Korean undiagnosed diseases program: lessons from a one-year pilot project

**DOI:** 10.1186/s13023-019-1041-5

**Published:** 2019-03-20

**Authors:** Soo Yeon Kim, Byung Chan Lim, Jin Sook Lee, Woo Joong Kim, Hyuna Kim, Jung Min Ko, Ki Joong Kim, Sun Ah Choi, Hunmin Kim, Hee Hwang, Ji Eun Choi, Anna Cho, Jangsup Moon, Moon Woo Seong, Sung Sup Park, Yun Jeong Lee, Young Ok Kim, Jon Soo Kim, Won Seop Kim, Young Se Kwon, June Dong Park, Younjhin Ahn, Joo-Yeon Hwang, Hyun-Young Park, Youngha Lee, Murim Choi, Jong-Hee Chae

**Affiliations:** 1 Department of Pediatrics, Pediatric Clinical Neuroscience Center, Seoul National University Children’s Hospital, Seoul National University College of Medicine, Seoul, Korea; 2 Department of Pediatrics, Department of Genome Medicine and Science, Gil Medical Center, Gachon University College of Medicine, Incheon, Korea; 3 Department of pediatrics, Division of Clinical Genetics, Seoul National University College of Medicine, Seoul, Korea; 4 Department of Pediatrics, Seoul National University Bundang Hospital, Gyeonggi-do, Korea; 5 Department of Pediatrics, SMG-SNU Boramae Hospital, Seoul, Korea; 6 Department of Neurology, Department of Neurosurgery, Seoul National University Hospital, Seoul, Korea; 7 Department Neurosurgery, Seoul National University Hospital, Seoul, Korea; 8 Department of Laboratory Medicine, Seoul National University Hospital, Seoul, Korea; 9 Department of Pediatric, School of Medicine, Kyungpook National University, and Kyungpook National University Hospital, Daegu, Korea; 10 Departmentof Pediatrics, Chonnam National University Medical School, Gwangju, Korea; 11 Department of Pediatrics, College of Medicine, Chungbuk National University, Cheongju, Korea; 12 Department of Pediatrics, School of Medicine, Inha University, Incheon, Korea; 13 Department of Pediatrics, Seoul National University College of Medicine, Seoul, Korea; 14 Division of Rare Diseases, center for Biomedical Science, Korea National Institute of Health, KCDC, Chungcheongbuk-do, Korea; 15 Center for Genome Science, Korea National Institute of Health, KCDC, Shungcheongbuk-do, Korea; 16 Department of Biomedical Sciences, Seoul National University College of Medicine, Seoul, Korea; 17 Division of Pediatric Neurology, Department of Pediatrics, Pediatric Clinical Neuroscience Center, Seoul National University Children’s Hospital, Seoul National University College of Medicine, 101 Daehakro Jongno-gu, Seoul, 110-744 Korea

**Keywords:** Rare disease, Undiagnosed disease program, Korea, Whole exome sequencing

## Abstract

**Background:**

The Korean Undiagnosed Diseases Program (KUDP) was launched in January 2017 as a one-year pilot project to address the increasing global interest in patients with undiagnosed rare diseases. The purpose of this paper is to summarize the project results and emphasize the unmet research needs among patients with undiagnosed rare diseases in Korea.

**Results:**

Patient enrollment, assessment, and diagnostic processes were determined by the KUDP clinical expert consortium. Patients followed a diagnostic workflow after being categorized into one of four groups: I) insufficient clinical information or lack of standard diagnostic processes; II) undiagnosed due to low disease awareness; III) clinically diagnosed but unconfirmed genetically due to genetic heterogeneities; or IV) unknown disease due to complex, atypical clinical presentations. After excluding two patients from group I, 97 patients were enrolled, including 10 in group II, 67 in group III, and 20 in group IV. Most of them (92 of 97, 94.8%) were pediatric patients (< 18 years old) and 59 (60.8%) were male. The primary symptoms for 80 patients (82.5%) were neurologic. During the one-year pilot study, 72 patients completed a diagnostic assessment including clinical and molecular genetic analyses; some patients also underwent pathological or biochemical analysis. Twenty-eight of these patients (28/72, 38.9%) achieved molecular genetic diagnosis. Thirteen patients were diagnosed based on traditional tests, including biochemical assay, single or targeted genetic analysis, and chromosomal microarray. We performed whole exome sequencing on 52 patients, among whom 15 (28.8%, 15/52) reached a final diagnosis. One new disorder was identified via international collaboration.

**Conclusions:**

Using an efficient clinical diagnostic workflow, this KUDP pilot study resulted in a fair diagnostic success rate, improving the potential for additional diagnoses and new scientific discovery of complex and rare diseases. KUDP also satisfied unmet needs for rare diseases with multisystem involvement, highlighting the value of emerging genomic technologies for further research into rare and still-undiagnosed conditions.

**Electronic supplementary material:**

The online version of this article (10.1186/s13023-019-1041-5) contains supplementary material, which is available to authorized users.

## Background

Rare disease (RD) is defined as one that affects fewer than 5 per 10,000 persons in the European Union (EU) or fewer than 200,000 persons in the United States (US). In Korea, the Rare Disease Act of 2016 defined RD as affecting fewer than 20,000 patients or for which the prevalence is unknown because of its rarity. There are countless RDs worldwide, with almost 8% of the general population suffering from a RD despite their relative rarity [1]. To date, more than 7000 RDs have been identified and their numbers have continued to increase, while there remain numerous unidentified diseases [2, 3]. Patients with a RD can spend more than 5–6 years reaching a diagnosis, while many remain undiagnosed [4, 5]. These circumstances cause excessive medical costs and long-term social burdens, as well as loss of well-being among both patients and their families. Thus, undiagnosed RDs are a global challenge that must be overcome.

Global networks, funds, and new technology development will be required to meet these goals. Fortunately, investigations are improving through the use of advanced genetic technologies, including next-generation sequencing (NGS) and growing international collaboration. Many countries have initiated projects on undiagnosed RDs, including the Undiagnosed Diseases Program (UDP) by the US National Institutes of Health (NIH), the Solving the unsolved rare diseases (Solve-RD) program funded by the European Commission, the Finding of Rare Disease Genes (FORGE) project in Canada, and the Initiative on Rare and Undiagnosed Diseases (IRUD) project in Japan [4, 6–8]. While Undiagnosed Diseases Network International (UDNI) was established with international effort, UDP-WA in Australia was clinically implemented within the public health system in 2016 [9]. For several years, the Korea National Institute of Health (KNIH) of the Korea Centers for Disease Control and Prevention (KCDC) have supported the genetic diagnosis of patients with some RDs. Recently, the KNIH added to their supporting policy all “undiagnosed” or “unknown” RDs and launched KUDP. The first step in this process was a one-year pilot project starting in January 2017 to determine the Korean context for undiagnosed patients and to plan a sustainable KUDP. Herein, we summarize the results of this one-year pilot project and emphasize the importance of a sustainable KUDP.

## Methods

### Study approval and preparation of the project

The pilot project was initiated and supported by the KNIH. Seoul National University Children’s Hospital functioned as the main center and supervised this process. Three regional hospitals were also involved to recruit and evaluate patients. The expert consortium consisted of more than 20 specialists covering nearly all divisions of pediatrics and related departments, such as pediatric neurology, cardiology, nephrology, hematology, surgery, as well as adult neurology, rheumatology, endocrinology, ophthalmology, otology, orthopedics, clinical genetics, laboratory medicine, and bioinformatics, across six institutes. Patients were enrolled via two methods: a referral letter from local clinicians, and by presenting directly to a central hospital clinic. Written informed consent for DNA preparation and detailed tests was obtained from all enrolled patients or their legal representatives. Procedures were approved by the Institutional Review Board (IRB) of Seoul National University Hospital (IRB No. 1101-110-353, 1406-081-588, 1511-099-722, 1408-010-599).

### Diagnostic workflow

A total of 99 patients were referred for the study. All but two of these visited the central hospital. The others were referred by local clinicians via recruitment letter. The expert consortium classified each patient into one of four groups: I) insufficient clinical information or lack of standard diagnostic processes; II) undiagnosed due to low disease awareness; III) genetically undiagnosed due to genetic and clinical heterogeneities; or IV) unknown disease due to complex, atypical clinical presentations. Two patients who were referred by letter were classified into group I and could not be recruited into the project because of a lack of clinical information and standard evaluation for suspicious diagnoses. The remaining 97 patients were finally enrolled and underwent a serial diagnostic process based on decisions by the clinical expert consortium. The first-tier test included chromosomal microarray, enzyme assay, single-gene sequencing, and targeted multi-gene panel. Whole exome sequencing (WES) was conducted as a first diagnostic test as well as a second-tier test, in some cases based on the decision of the expert consortium. Muscle biopsy was also performed in two patients with suspicious primary muscle disease. In cases undiagnosed after the first-tier test, the expert consortium reviewed each one and decided on the next step, including performing a second-tier genetic test or connecting to nationwide and/or international scientific research networks. Detailed procedures, including DNA extraction, for each test have been previously described [10–12].

## Results

### Patient characteristics

**Fig. 1:**
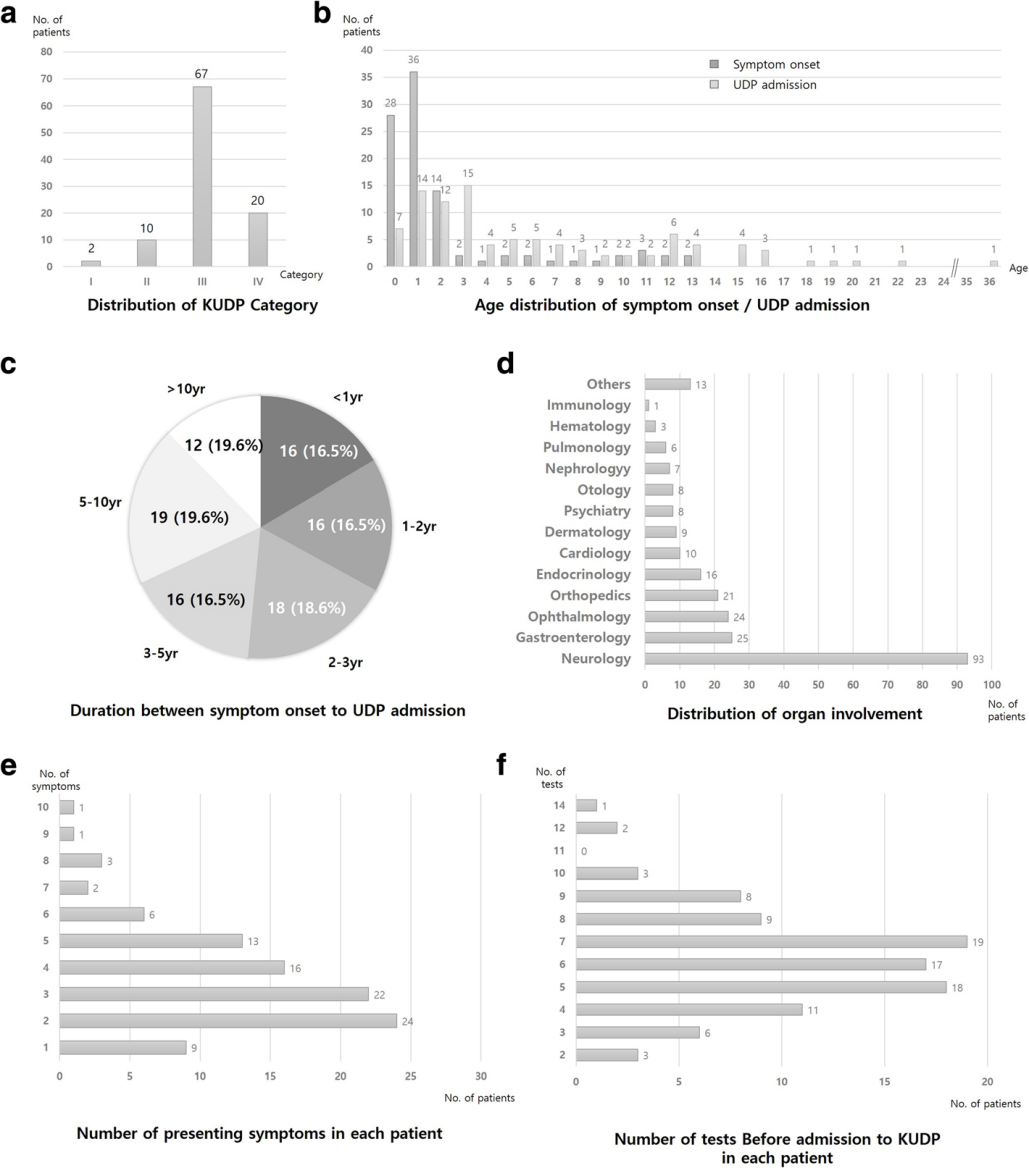
Characteristics of the enrolled patients. **a** Number of patients in each category. **b** Age distribution at initial symptom onset and KUDP admission. **c** Time between initial symptom onset and KUDP admission. **d** Distribution of organ involvement. **e** Number of presenting symptoms per patient. **f** Number of completed tests for each patient before KUDP admission

Demographic data for the 97 patients are described in Table 1 and Fig. 1. Ninety-two (94.8%) were children (under 18 years old) and 59 (60.8%) were male. Patients under 5 years of age accounted for over half of the patients, whereas there were only five patients over 18 years. Patients’ first hospital visit was when they were on average 2.5 years old (0–18.4 years). Seventy-six patients (76/97, 78.4%) first accessed medical services within 3 months of symptom recognition. On their diagnostic journey, these patients visited an average of 2.5 clinics, including 1.6 tertiary centers, such as university-based major medical centers, where they had an average of 6.2 types of tests (range 2–14). About 70% of patients (68 of 97) had one or more genetic tests, including chromosomal microarray, single gene sequencing, designed target gene panel sequencing, and clinical exome sequencing, which uses the TruSight One Sequencing Panel to target more than 4800 genes associated with specific clinical phenotypes. Despite exhaustive diagnostic efforts, they remained undiagnosed for an average of 5.3 years (0–17.7 years). More than half of the patients (67/97, 69.1%) were classified into group III, 10 into group II, and 20 into group IV. More than 90% of these patients (93/97, 95.9%) complained of one or more neurologic manifestations, including developmental delay, intellectual disability, intractable seizures, involuntary movements, or muscle weakness. All but nine patients (88/97, 90.7%) had more than one presenting symptom in different organs: gastrointestinal problems including growth failure in 25 (25.8%); ophthalmologic symptoms in 24 (24.7%); and skeletal involvement in 21 (21.6%).

**Table 1:** Enrolled patient demographics

	Number of patients (total *n* = 97)
Sex (male:female)	59:38
Mean age of symptom onset (years)	2.1 (0–13.8)
Mean age at first medical service (years)	2.5 (0–18.4)
Mean age at KUDP admission (years)	6.7 (0–36.0)
Mean Number of tests before the project	6.2 (2–14)
Number of visited clinics before the project admission (n, (%))	
1	7 (7.1)
2	52 (52.5)
3	26 (26.3)
> 4	14 (14.1)
Clinical diagnosis (n, (%))	
Neurodevelopmental	54 (55.7)
Congenital multiple anomaly	7 (7.2)
Metabolic	20 (20.6)
Neuromuscular	11 (11.3)
Others	5 (5.2)

### Diagnostic success rate

After KUDP admission, patients underwent individualized tests by stages. The overall schematic diagnostic process and its result are shown in Additional file 1: Figure S1. Among the 97 patients, 72 (74.2%) completed one or more diagnostic process and 28 (28/72, 38.9%) had confirmative diagnosis. Ten patients in group II had traditional tests, including single-gene sequencing, single enzyme assay, and multi-gene panel sequencing, based on clinical suspicions. Among these, eight patients (8/10, 80%) received confirmative diagnoses. In most cases, patients in groups III and IV had WES as their first- or second-tier tests, although five patients achieved a molecular diagnosis through traditional tests. Diagnostic yield according to each modality is described in Table 2. Traditional tests, including biochemical assay, single or targeted genetic analysis, and chromosomal microarray, led to accurate molecular diagnosis in 13 of the 31 completely analyzed tests (41.9%). WES indicated 28.8% of the diagnostic yield: 25.0% for proband only and 35.0% for trio analysis. None of these patients had dual diagnoses. In addition, three candidate genes that had not been previously reported in human diseases were found. We initiated a functional study of one candidate gene and used web-based matchmaking tools for the other two [13]. Detailed genotypes and phenotypes for each case with genetic confirmation are described in Table 3.

**Table 2:** Diagnostic tests and results

	Total number of test performed (n)	Analysis complete (n)	Diagnosis confirmed (n)	Diagnostic yield (%)
Traditional test				
Chromosomal microarray	14	12	2	16.7
Enzyme assay	2	2	2	100.0
Single gene test	10	9	4	44.0
mtDNA sequencing	1	1	1	100.0
Target Gene panel sequencing	7	7	4	57.1
Exome, proband	54	32	8	25.0
Exome, trio	40	20	7	35.0

*mtDNA* mitochondrial DNA

**Table 3:** Phenotypes and genotypes of positive cases

No.	Gender/age	Clinical presentation	Diagnostic test	Genotype	Associated disease (MIM number.)
1	M/7.9	Global developmental delay, microcephaly, facial dysmorphism, photosensitivity	WES, proband	*ERCC5*, c.1627-2A > G	Cockayne syndrome (MIM #278780)
2	F/3.9	Developmental delay, myopathic face	Single gene	*FKTN*, c.165 + 835 T > G/c.49A > C	Muscular dystrophy-dystroglycanopathy (MIM #253800)
3^a^	M/5.4	Gait abnormality	Enzyme assay	*ARSA*, c.1107 + 1delG/c.919A > C	Metachromatic leukodystrophy (MIM #250100)
4	M/17.8	Seizure gait abnormality, intellectual disability	WES, proband	*SLC2A1*, c.276-1G > A	GLUT1 deficiency syndrome 2, childhood onset (MIM #612126)
5^a^	F/2.2	Global developmental delay	Enzyme assay	*GLB1*, c.1343A > T/c.517_519del	GM1-gangliosidosis, type I (MIM #230500)
6	M/1.6	Congenital hypotonia	Single gene	*MTM1*, c.566A > G	Myotubular myopathy, X-linked (MIM #310400)
7	M/15.3	Global developmental delay, seizure	CMA	16p22.3 (29673954–30,119,759) 0.44 Mb deletion	
8	M/9.5	Paroxysmal dyskinesia	Singe gene	*PRRT2*, c.649dupC	Episodic kinesigenic dyskinesia (MIM #128200)
9	M/14.4	Progressive dystonia, dysarthria, dysphagia	Single gene	*ATP1A3*, c.2305A > C	Dystonia-12 (MIM #128235)
10	M/9.0	Congenital hypotonia, motor developmental delay, joint laxity	Targeted multi-gene panel	*COL6A1*, c.850G > A	Ullrich congenital muscular dystrophy, 1 (MIM #254090)
11	M/3.3	Intrauterine ventriculomegaly, developmental delay, hypotonia	Targeted multi-gene panel	*POMGNT1*, c.1702T > C/c.9445dupT	Muscular dystrophy-dystroglycanopathy, type A, 3 (MIM #253280)
12	F/2.9	Microcephaly, global developmental delay, hearing loss	CMA	17p13.1 (7138534–8,151,307) 1 Mb deletion	
13	M/3.0	Global developmental delay, early onset seizure	Targeted multi-gene panel	*SCN8A*, c.3820G > A	Epileptic encephalopathy, early infantile, 13 (MIM #614558)
14	M/9.8	Global developmental delay, seizure, abnormal skin lesion	WES, trio	*RAB11B*, c.64G > A	Neurodevelopmental disorder with ataxic gait, absent speech, and decreased cortical white matter (MIM #617807)
15	M/6.0	Global developmental delay, dysmorphic face, sparse hair, anhydrosis, dental anomaly	WES, proband	*EDA*, c.1045G > A	Ectodermal dysplasia 1, hypohidrotic, X-linked (MIM #305100)
16	M/13.8	Developmental regression, seizure, dystonia	WES, proband	*CLN6*, c.806C > T/c.184C > T	Ceroid lipofuscinosis, neuronal, 6 (MIM #601780)
17	F/11.9	Global developmental delay, stereotyped hands movement, seizure	WES, trio	*SLC6A1*, c.1070 C > T	Myoclonic-atonic epilepsy (MIM #616421)
18	F/10.8	Global developmental delay, dysmorphic face	WES, proband	*NAA10*, c.247C > T	Ogden syndrome (MIM #300855)
19	F/14.9	Ataxia	WES, trio	*POLR1C*, c.698insAAG/c.713A > G	Leukodystrophy, hypomyelinating, 11 (MIM #616494)
20	F/7.5	Recurrent infection, asthma, thrombocytopenia	WES, trio	*NFKB2*, c.2593_2594del	Immunodeficiency, common variable, 10 (MIM #615577)
21	M/3.3	Neonatal seizure, developmental delay	WES, proband	*ALDH7A1*, c.1279G > C	Epilepsy, pyridoxine dependent (MIM #266100)
22	F/8.8	Early onset seizure, global developmental delay, dysmorphic face	WES, trio	*DNM1*, c.709C > T	Epileptic encephalopathy, early infantile, 31 (MIM #616346)
23	F/3.2	Progressive respiratory distress	WES, proband	*SFTPC*, c.203T > A	Surfactant metabolism dysfunction, pulmonary, 2 (MIM #610913)
24	F8.3	Ataxia, seizure, progressive muscle weakness	mtDNA sequencing	m.8344A > G	
25	M/22.3	Progressive scoliosis, scapular deformity	Targeted multi-gene panel	*GAA*, c.T1316A/c.G2238C	Glycogen storage disease II (MIM #232300)
26	M/12.8	Global developmental delay, failure to thrive, congenital heart disease	WES, trio	*POC1A*, c.241C > T/c.239C > T	Short stature, onychodysplasia, facial dysmorphism, and hypotrichosis (MIM #614813)
27	F/8.6	Congenital hypotonia, failure to thrive, bilateral hip dislocation, imperforated anus, congenital heart disease	WES, trio	*HSPA9,* c.383A > G/c.884_885del	Even-plus syndrome (MIM #616854)
28	M/15.7	Tip toeing, slurred speech, pathologic reflexes	WES, proband	*C19orf12*, c.294del	Neurodegeneration with brain iron accumulation, 4 (MIM #614298)

*WES* whole exome sequencing, *CMA* chromosomal microarray

^a^Consequence Sanger sequencing for *ARSA* and *GLB1* was performed after final diagnosis through enzyme assay for genetic counseling

### Illustrative cases

#### Ending the diagnostic odyssey

A 10-month-old girl admitted to KUDP initially presented with developmental delay from the age of 5 months. She had been seen at two different tertiary care neurology clinics and had undergone extensive testing, including repeat brain magnetic resonance imaging (MRI), metabolic screening, and diagnostic exome sequencing. These comprehensive evaluations failed to identify genetic defects, although she was identified as having developmental delay with brain malformation. After KUDP admission followed by reevaluation at an expert clinic, additional findings such as myopathic face and elevated serum CK level (6275 IU/L, normal range 20–270 IU/L) were identified. The expert consortium suspected alpha-dystroglycan-related congenital muscular dystrophy, including Fukuyama type, and fukutin gene analysis was performed. Compound heterozygous mutations in *FKTN*, c.165 + 835 T > G (deep intronic mutation, founder mutation in Korean population) and c.49A > C, were identified as a confirmative diagnosis [14].

In another case, a 12-month-old girl had been evaluated for developmental delay at two other tertiary hospitals. Brain MRI had revealed mild brain atrophy and delayed myelination, and laboratory tests including metabolic screening were unremarkable except for slightly elevated liver enzymes. She was admitted to KUDP and reevaluated by the expert consortium, which recognized her developmental arrest as being followed by regression and noticed diffuse hypomyelination on her brain MRI. We then performed a skeletal survey, which indicated dysostosis multiplex such as inferior beaking of the lumbar spine. Suspecting GM1-gangliosidosis, enzyme assay for β-galactosidase was ordered, which revealed markedly decreased activity (1.0 nmol/hr./mg protein, normal range 80–140 nmol/hr./mg protein). We also performed Sanger sequencing for *GLB1* and identified compound heterozygous mutations, c.517_519delCTC and c.1343A > T.

#### Correction of misdiagnosis

**Fig. 2:**
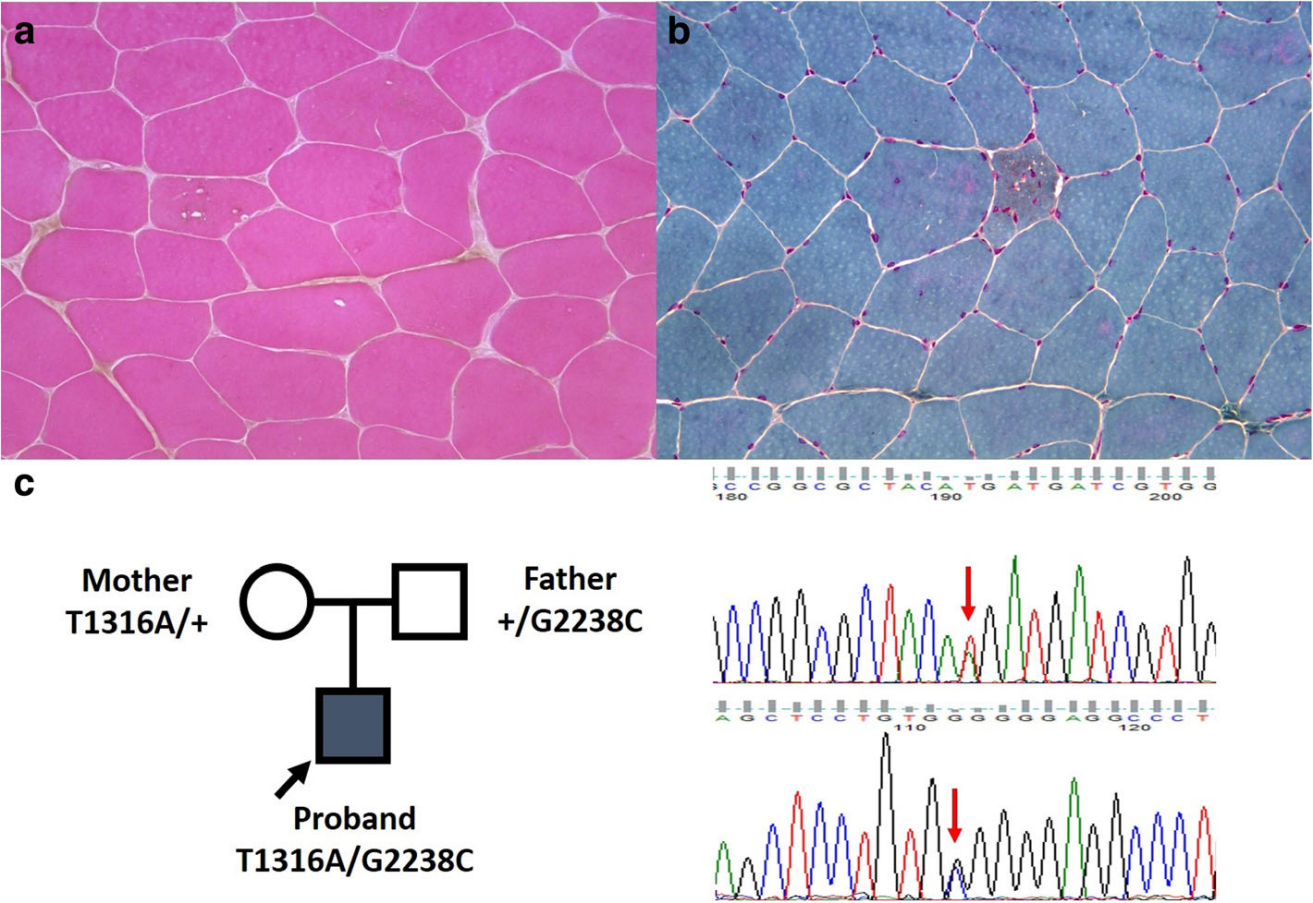
Pathology findings and mutation profiles of patient with Pompe disease. **a**, **b** Muscle pathology with hematoxylin and eosin staining and modified Gomori trichrome staining, indicating small angulated muscle fiber with rimmed vacuole, respectively. **c** Pedigree with mutation profiles and result of GAA sequencing for patient

A 13-year-old boy with progressive lordosis, asymmetric scapular deformity was admitted to KUDP. For 9 years prior to admission, his clinical diagnosis was facioscapulohumeral muscular dystrophy, although there were neither abnormalities in his genetic analysis nor specific findings of muscle pathology. After KUDP admission, we reanalyzed the patient’s phenotype; under the clinical diagnosis of another type of primary muscle disease, targeted multi-gene panel sequencing for muscle disease was performed, revealing compound heterozygous mutations of *GAA* (c.1316T > A and c.2238G > C). The boy was finally diagnosed with late-onset Pompe disease, and started enzyme replacement therapy. After molecular diagnosis, we again reviewed his muscle pathology and, interestingly, noticed very few muscle fibers with fine, small purple-colored granules, a pathognomonic finding suggesting Pompe disease (Fig. 2). This case clearly demonstrated the clinical utility of NGS in atypical presentation of known genetic disorder. In addition, it highlights the importance of appropriate treatment with accurate diagnosis, which can reverse the clinical course.

#### Discovery of a new disease via international collaboration

An 8-year-old boy was admitted to KUDP with developmental delay and cryptorchidism since birth, and progressive melanocytic nevus with skin pigmentation since the age of 1 year. His brain MRI revealed myelination delay and corpus callosum hypogenesis. As there was no definite clinical diagnosis, trio WES was performed, revealing a de novo variant in *RAB11B*, c.64G > A. Because *RAB11B* had never been reported in a human as a disease-causing gene, we shared the phenotype and genomic data with other international groups through the online matchmaking service and thereby confirmed that the genetic defect disturbing the GTP/GDP binding pocket of RAB11B caused this specific neurodevelopmental syndrome [15].

## Discussion

KUDP was launched in January 2017 as a one-year pilot project, considering the growing international interest in undiagnosed patients with RDs, to determine the feasibility and usefulness of a sustainable UDP in Korea and a medical system for rare disease diagnostics. Recently emerging genomic technology and international collaboration made it possible to increase insights into various and complex RDs and their phenotypes. Comparable to previous studies, this report shows that more than three-quarters of recruited patients were children younger than 18 years old who had neurologic symptoms [4, 16–19]. Only limited data have been published detailing patients’ struggles for a definite RD diagnosis. According to some studies, patients can spend 5–6 years awaiting a molecular diagnosis, after multiple visits to clinics and 1–12 diagnostic tests (average 3–5) [17, 19]. In our study, patients visited on average 2.5 clinics (range, 1–5) including 1.6 tertiary hospitals and had 6.2 tests prior to KUDP admission. We also evaluated medical accessibility, represented by the time from symptom onset to their first hospital visit, which was an average of 6 months, with 90 of 97 patients (92.8%) able to access tertiary hospital care within 12 months. Based on the Korean medical referral and insurance system, accessibility of the tertiary hospitals (e.g., major medical centers including the KUDP central hospital) was simple. Therefore, we facilitated two different routes to KUDP admission: 1) a referral letter from clinicians and 2) a direct visit for the patient to the KUDP central hospital. All included patients enrolled in KUDP using the latter route, which likely had advantages, including that experts could examine the patients directly and decide whether to enroll them or not within a short period. Considering the Korean medical referral and insurance system, we would want to maintain those two separate routes to KUDP admission, although we expect a gradual increase in enrolled patients through a nationwide network from regional clinics. We classified patients into four categories (I–IV). Category II, undiagnosed due to low awareness, consisted of undiagnosed patients who had received extensive diagnostic workup at other tertiary university-based medical centers. We enrolled these patients in the “pilot” project to evaluate their rare disease diagnostic status within the current health care system and to develop a nation-wide network for successful and effective development of diagnostic pathway of the KUDP. Henceforth, we plan to exclude patients in category II from the main UDP project and refer them back to their local network hospital, along with guidelines for appropriate diagnostics. We also plan to expand the adult patients included in the program and focus on undiagnosed patients in categories III and IV, followed by related functional research studies.

Among the 97 enrolled patients, one or more tests with analyses were completed in 72, and 28 patients received a final diagnosis (diagnostic success rate: 28/72, 38.9%) during 1 year (from January 2017 to December 2017). Traditional tests including chromosomal microarray, enzyme assay, single-gene analysis, mitochondrial DNA sequencing, and multi-gene panel accounted for 26.6% (34/128) of the tests performed, which led to confirmative diagnosis in 13 patients. For the 14 patients who remained undiagnosed after traditional tests, WES was usually performed as a second-tier test. This was also useful as a first diagnostic step in relatively well-known genetic disorders with multiple causative genes or missed patients with complex and atypical presentation. As for exome sequencing, we had diagnostic success in 15 of 52 patients whose analyses were completed (diagnostic yield: 15/52, 28.8%). This rate is similar to previous studies with WES, which has shown rates of 25–51.9% [17–21]. Our total success rate was 38.9%, and the diagnostic yield within categories III and IV was 31.7% (20 of 63), similar to previous studies [22]. In contrast, the success rate for the WES in the first year of this pilot program was relatively lower, possibly because of incomplete WES within the proband. Twelve patients were awaiting additional trio exome sequencing and were postponed because of budget restrictions (the initial budget was 130,000,000 Korean Won/year). Work on these patients will soon resume as part of the main project; thus, the success rate is likely to further increase.

Despite some differences and limitations compared with other undiagnosed patient programs, the diagnostic workflow through the KUDP pilot project was quite efficient and resulted in a high yield rate through use of frequent expert consortium meetings and well-organized genetic test strategies, the latter of which ranged from traditional to advanced tests based on the patients’ clinical features. The importance of appropriate diagnostic tests, including detailed history taking and traditional tests, has been identified previously [22]. Furthermore, we verified some likely pathogenic variants in novel genes that have not previously been reported in human disease. We registered these variants on an online international match program to find that one had been published by another study group as a new disease discovery [15]. We also started functional validation of another three genes either independently or through international collaboration and further scientific achievements are thus expected (data now shown). We anticipate that the diagnostic yield may increase following completion of all diagnostic processes and additional functional studies with international collaborations.

Despite this, more than half of the cases reported here may remain undiagnosed after all factors have been considered, a rate similar to undiagnosed patient programs in other countries [17, 18, 23]. Further research is therefore needed for those patients, combined with advanced bioinformatics and genomic technologies such as RNA (transcriptome) sequencing and whole-genome sequencing, and more active international data sharing and collaborations [20].

Overall, the one-year pilot KUDP project demonstrated full feasibility and established the need for planning a sustainable KUDP. The cases illustrated here support the program’s necessity in terms of correcting misdiagnosis, ending patients’ diagnostic odyssey, and facilitating precise genetic counseling and therapeutic options for patients.

## Conclusions

We have summarized the results of the one-year pilot Korean Undiagnosed Diseases Program. Using an efficient diagnostic process, we achieved a successful diagnostic rate of 38.9% and clearly illustrated the need for an ongoing, sustainable program for undiagnosed rare diseases in Korea.

## Additional file

Additional file 1: **Figure S1.** Schematic diagram of diagnostic workflow of all patients. (JPG 213 kb)

## Abbreviations

IRB: Institutional Review Board
KCDC: Korea Centers for Disease Control and Prevention
KNIH: Korea National Institute of Health
KUDP: Korean Undiagnosed Diseases Program
NGS: Next-generation sequencing
WES: Whole exome sequencing
