# Supplementary figures and images for: The Korean undiagnosed diseases program: lessons from a one-year pilot project
Source: Orphanet J Rare Dis. 2019 Mar 20;14:68. doi: 10.1186/s13023-019-1041-5 (PMC6427886; doi:10.1186/s13023-019-1041-5)

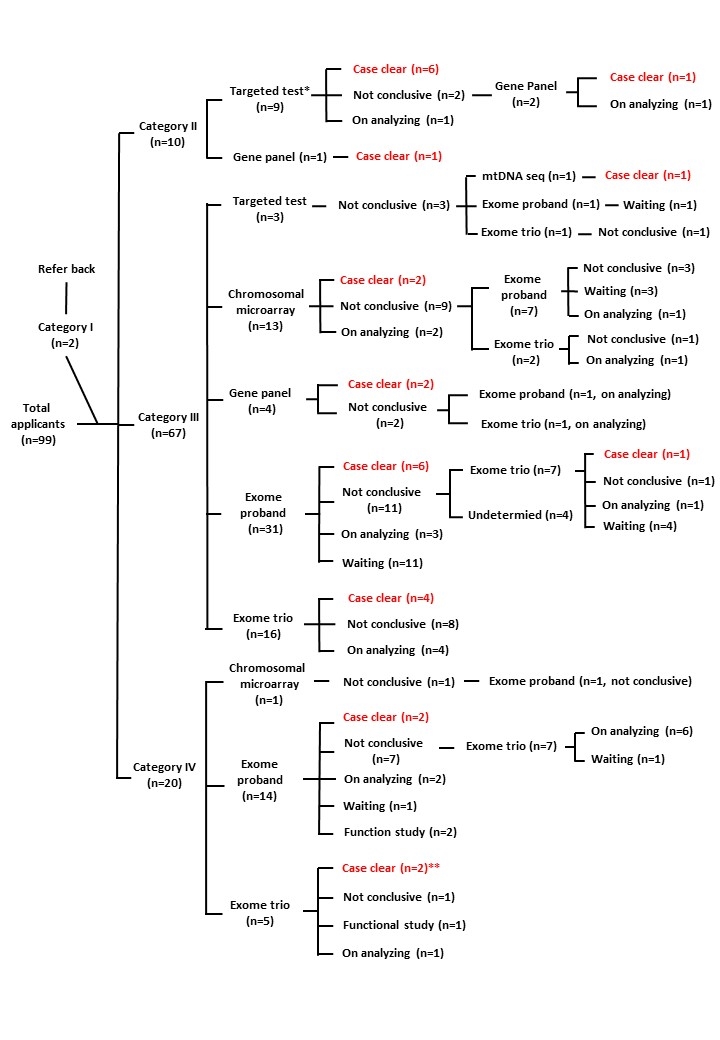

Supplement: Supplementary file 1 — Figure S1. Schematic diagram of diagnostic workflow of all patients. (JPG 213 kb) [file 13023_2019_1041_MOESM1_ESM.jpg]
